# A New Laparoscopic Surgical Procedure to Achieve Sufficient Mesorectal Excision in Upper Rectal Cancer

**DOI:** 10.1155/2011/708439

**Published:** 2011-10-20

**Authors:** Seiji Ohigashi, Takashi Taketa, Kazuki Sudo, Hironori Shiozaki, Hisashi Onodera

**Affiliations:** Department of Gastroenterological Surgery, St. Luke's International Hospital, 9-1 Akashi-cho, Chuo-ku, Tokyo 104-8560, Japan

## Abstract

*Objective*. Mesorectal excision corresponding to the location of a tumor, termed tumor-specific mesorectal excision (TSME), is commonly performed for resection of upper rectal cancer. We devised a new laparoscopic procedure for sufficient TSME with rectal transection followed by mesorectal excision. *Operative Technique*. After mobilization of the sigmoid colon and ligation of inferior mesenteric vessels, we dissected the mesorectum along the layer of the planned total mesorectal excision. The rectal wall was carefully separated from the mesorectum at the appropriate anal side from the tumor. After the rectum was isolated and transected using an endoscopic linear stapler, the rectal stump drew immediately toward the anal side, enabling the mesorectum to be identified clearly. In this way, sufficient TSME can be performed easily and accurately. This technique has been successfully conducted on 19 patients. *Conclusion*. This laparoscopic technique is a feasible and reliable procedure for achieving sufficient TSME.

## 1. Introduction

**Figure 1:**
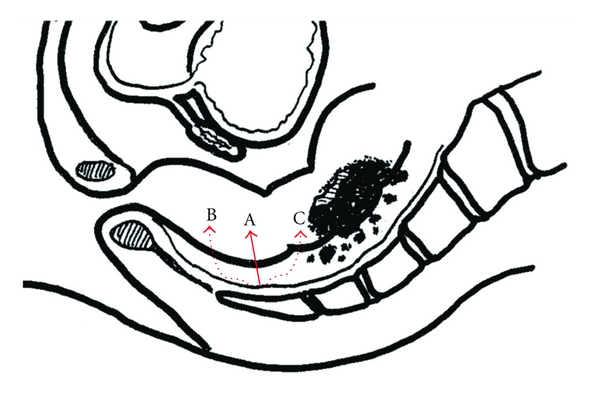
(A) Ideal resection line of the mesorectum. (B) and (C) Inappropriate resection line of the mesorectum.

Total mesorectal excision (TME) is recognized as an extremely important surgical technique for the prevention of local recurrence of rectal cancer [1–3]. On the other hand, TME is not necessarily applicable in every case of rectal cancer: for upper rectal cancer, mesorectal excision for limited lengths of 5 cm from a tumor toward the anal side is widely conducted, and this method is reportedly associated with adequate rates of cure [4, 5]. This technique is referred to as partial mesorectal excision (PME), but rather should be called tumor-specific mesorectal excision (TSME) reflecting its correspondence to the localization or T-stage of the tumor [5]. In a narrow pelvic cavity, performing sufficient TSME is difficult, and there is a risk of local recurrence when TSME is inadequate [6–8]. Whether surgery is performed laparoscopically or via a conventional open route, TSME is usually conducted obliquely to the anal side, introducing unnecessary rectal resection which may lead to postoperative bowel malfunction [9]. In addition, there is of the potential for slippage of the TSME between the right and left sides of the rectal wall. Particularly in the case of laparoscopic surgery, straight and sharp dissection of the mesorectum is difficult to perform and the dissection line is likely to be in zigzags. Of course, TSME shifting toward the oral side from the starting line is inappropriate and should be strictly avoided in order to prevent local recurrence (Figure 1).

To overcome these difficulties, we have introduced a technique to transect the rectum before resection of the mesorectum during conventional open surgery [10]. This technique assures an adequate distance between the tumor and resected stump on the anal side and sufficient TSME corresponding appropriately to the T-stage of the tumor. This technique is also applicable to the laparoscopic approach, which we report here.

## 2. Operative Technique

The most appropriate indication for this technique is the treatment of upper rectal cancer of stage T-2 and higher. Here, we report details of the techniques used in our laparoscopic procedure.

**Figure 2:**
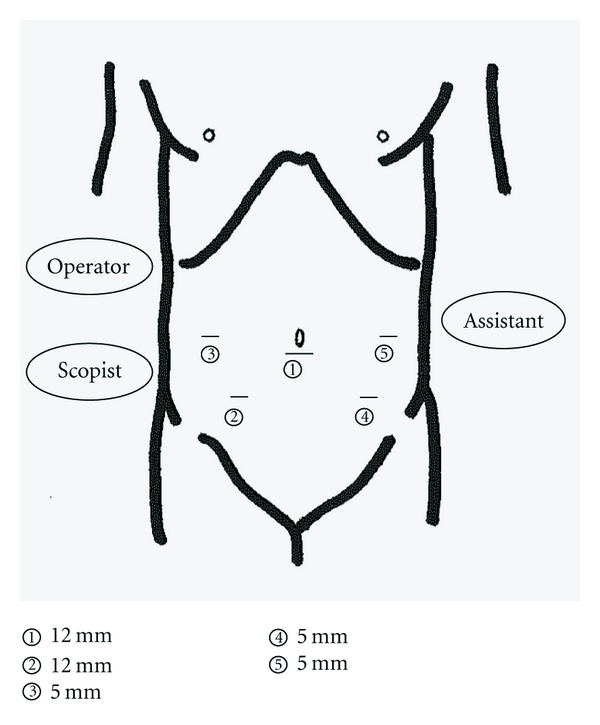
A total of five trocars are used.

A trocar for laparoscope was inserted just beneath the umbilicus; we used four working ports as shown in Figure 2. Surgery commenced with mobilization of the sigmoid colon with a preference for the approach from median to lateral [11]. After having freed sigmoid colon thoroughly from the retroperitoneum, the inferior mesenteric artery (IMA) was ligated for lymph nodes dissection. The inferior mesenteric vein was ligated at the level of the IMA origin.

Next, we started to dissect the mesorectum at the posterior site. After visually confirming the left and right hypogastric nerves, we dissected the mesorectum in the layer just above the nerve leaving the nerve intact as if to draw a semicircular line. In case of TSME, there was no need to dissect as deeply as to the point where the levator ani was exposed, and thus, we aimed to dissect several centimeters more toward the anal side from the scheduled mesorectal excision line. Then, we proceeded to the anterior site. In cases with anastomosis planned under the peritoneal reflection, we tried to dissect the dorsal site of Denonvilliers' fascia; however, if the tumor was located at the anterior wall of the rectum, Denonvilliers' fascia should be deliberately resected with the rectum [12]. Finally, lateral attachments on both left and right sides were resected to accomplish mobilization of the rectum enveloped within the fascia propria recti.

**Figure 3:**
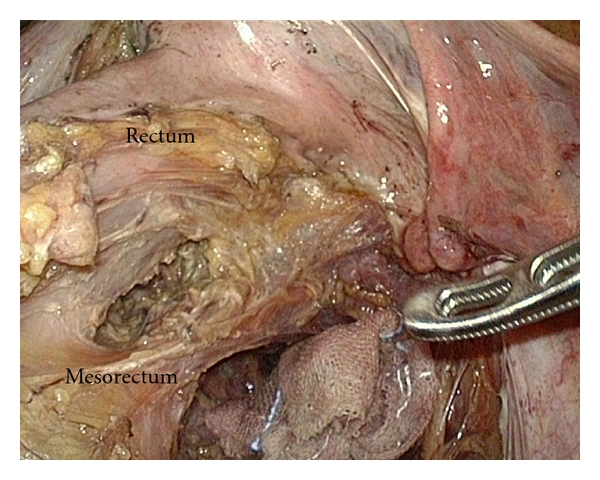
Separation of the rectum is started from the right side of the mesorectum.

After the above procedure was completed, we moved on to separation of the rectal wall from the mesorectum with an adequate distance from the tumor in accordance with its T-stage. Prior to the operation, tattoo in black ink was applied to the nearest site of the tumor endoscopically. In addition, during the operation, we directly painted the rectum about 5 cm from the tattoo toward the anal side with crystal violet. The separation was usually begun from the right wall of the rectum at the marked site. With laparoscopy, every vessel could be observed precisely because of its magnifying effect [13] and use of the curved shears (Harmonic Ace; Ethicon Endosurgery Inc.), enabling safe and rapid resection of the vessels. In order to provide enough space to insert an endoscopic linear stapler, only the mesorectum just underneath the rectal wall was excised for about 3 cm in width along the rectal tube (Figure 3). The mesorectal excision should be conducted from the right side as much as possible. Lastly, the mesorectum just underneath the rectal wall was excised in order to separate the rectal wall completely from the mesorectum. 

**Figure 4:**
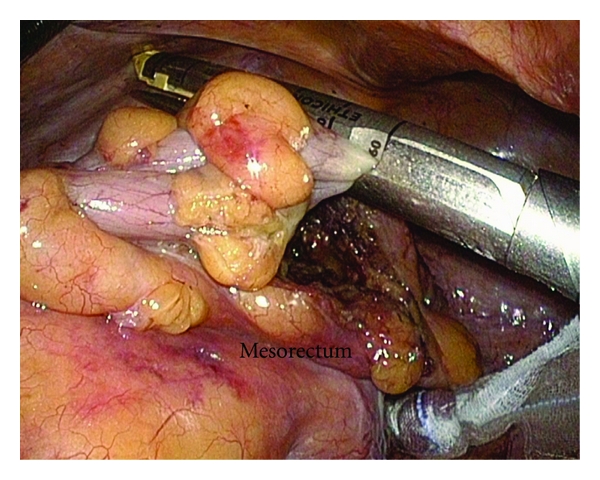
After the rectum is completely separated from the mesorectum, the rectum is transected using linear staplers.

**Figure 5:**
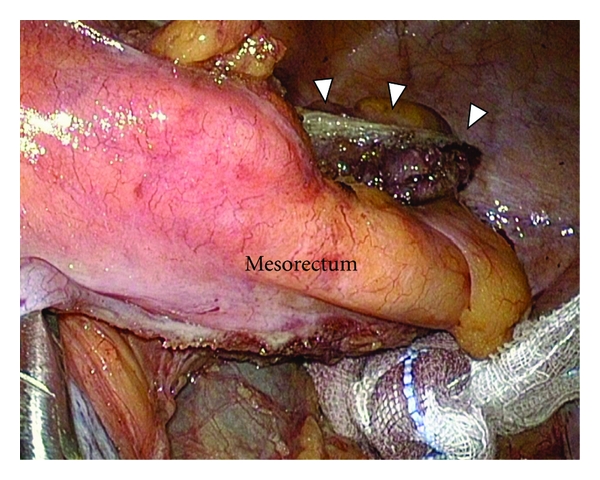
After transection of the rectum, the mesorectum can be observed clearly. The arrow heads show the distal rectal stump.

**Figure 6:**
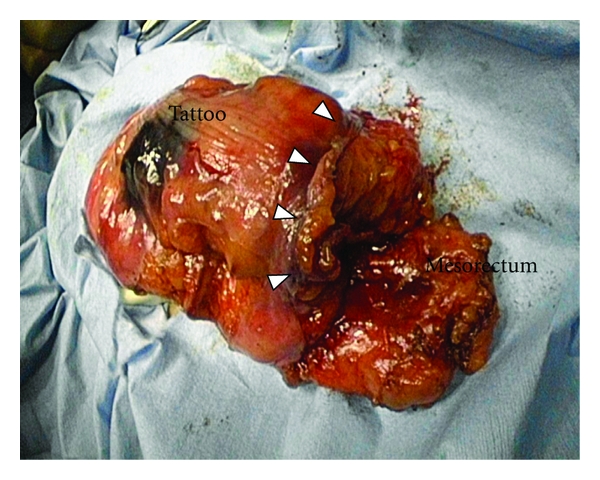
The mesorectum is sufficiently resected with the specimen. The arrow heads show the proximal rectal stump.

After the rectal wall was sufficiently separated from the mesorectum, the rectum was closed by a clamped forceps to irrigate inside the rectum with 2 liters of saline via the anus. Then, the rectum was transected using an endoscopic linear stapler (Figure 4). In most cases, the rectum was transected by the first firing, because only the rectal wall without mesorectum had been dissected. When the rectum was transected, the distal rectal stump was drawn toward the anal side; moreover, by pulling the proximal rectum toward the cranial side, several centimeters of mesorectum that did not adhere to the rectum could be confirmed visually (Figure 5). This area of mesorectum was then resected using the Harmonic Ace. This made it easy to sharply and precisely excise the mesorectum along a straight line from the distal rectal stump on the anal side toward the sacrum in a short period of time. According to the specimen, the mesorectum was resected as if a large volume of it were drawn out of the rectal stump, showing satisfactory TSME (Figure 6).

Lastly, a small incision of 3-4 cm was made above the pubic bone, and the specimen was transected outside the abdomen. After having inserted the anvil head of a circular stapler into the sigmoid colon, intracorporeal anastomosis was performed using double stapling techniques. As long as the donuts were checked and a complete ring was confirmed after the anastomosis, no drain was placed, and no diverting stoma was performed.

## 3. Results

Laparoscopic TSME using this technique was conducted on 19 patients from April 2008 to March 2011. Tumor localization was the distal sigmoid colon in 5 patients and the upper rectum in 14 patients. There were 10 men and 9 women; mean age was 67 years (range 46–79). Mean blood loss was 86 ml (range, 15–320 ml) and mean operating time was 3 hours and 47 minutes (range, 2 hours 45 min–5 hours 11 min). There was no incidence of rectal wall injury during the separation of the rectum from the mesorectum. The rectum was successfully transected by an endoscopic linear stapler in one attempt in 17 out of 19 patients. Firing was required twice for 2 patients. Postoperative anastomotic leakage occurred in one patient and diverting colostomy was performed. The average distance from the rectal stump to the distal mesorectum in freshly resected specimen was 20 mm (range 8–30 mm), indicating satisfactory TSME.

## 4. Discussion

The main goal of TME is to resect the mesorectum, especially the anal side of small metastatic lesions termed tumor deposits and the area surrounding the mesorectum *en block* [1, 2]. This technique contributes largely to reducing postoperative local recurrence of colon cancer [3, 8]. Generally, the mesorectum becomes thinner as it gets closer to the levator ani, and in case of TME for lower rectal cancer, there is often no need for special treatment of the mesorectum. However, in case of TSME, the primary tumor site is either in the rectosigmoid or upper rectum and the mesorectum at the scheduled resection line about 5 cm toward the anal side from the primary cancer is thick [5, 14]. Because of this, in the conventional procedure, the mesorectum is resected first, and, after having exposed the rectal wall, the rectum is transected. In a narrow pelvic cavity, it is not always easy to conduct appropriate mesorectal excision at an adequate distance from the tumor; the mesorectum is likely to be resected obliquely toward the anal side. In addition, it is difficult to sharply resect the mesorectum laparoscopically, and the resection tends to proceed in a zigzag line. Also, in an attempt to avoid injuries to the rectal wall during laparoscopic surgery, the mesorectal excision is likely to be insufficient. This is one factor leading to the repeated use of a linear stapler for transection of the rectum. Needless to say, there is increased risk of anastomotic leakage with repeated stapler firing [15, 16]. 

The merits of this procedure are as follows: (1) separating the rectum in advance allows rectal transection at the targeted line, leaving an adequate distance along the anal side; (2) the mesorectal excision is made easy and secure by a good visual field provided by the rectal transection; (3) there is more chance of transecting the rectum successfully of the linear stapler, because the rectal wall has already been separated. With regards to mesorectal excision especially, the mesorectum to be resected can be identified with a good visual field when a rectal stump draws toward the anal side after cutting off the rectum. At the same time, the proximal rectum is pulled toward the cranial side. The mesorectal excision after this is extremely easy, and the use of energy devices such as the Harmonic Ace makes it possible to conduct a sharp and straight linear excision of the mesorectum in a short period of time. The resected specimen also shows that the mesorectal excision is more sufficient by this method than when done in a conventional way. When the conventional method is performed, after transecting the rectum, a rectal stump sometimes slips into mesorectal fat, and we know by experience that by pushing in a shaft of circular stapler from the anus, the rectal stump that is forced out with the mesorectum can be finally identified. By this new method, however, because the mesorectum is sufficiently resected, a rectal stump can always be identified visually, and the shaft can be easily maneuvered. It also can be used as proof that the mesorectal excision has been sufficiently conducted. 

For surgical resection of rectal cancer, adequate mesorectal excision is important. Especially in case of upper rectal cancer, TME is not necessary; instead, TSME is sufficient [4, 5, 14]. Many surgeons may feel that it is not always easy to conduct TSME in a narrow pelvic cavity. The method presented here is helpful in lessening such burdens and is still compatible with the concept of TME, which places much emphasis on dissecting sufficient mesorectum on the anal side and removal of tumor deposits en block. Whether this method has any effect on decreasing the local recurrence rate is unknown; randomized controlled trials are needed to investigate this. In this paper, the authors would like to emphasize the merit of this method. As accuracy is an important factor in TSME, this method can provide a good visual field when the mesorectal excision is conducted.
